# Elevation of spermine remodels immunosuppressive microenvironment through driving the modification of PD-L1 in hepatocellular carcinoma

**DOI:** 10.1186/s12964-022-00981-6

**Published:** 2022-11-08

**Authors:** Hong-Xiang Shi, Chao Liang, Chao-Yan Yao, Zi-Xuan Gao, Jia Qin, Jin-Lan Cao, Ming-Zhu Zhang, Ying-Ying Li, Meng-Qing Wang, Hua Sun, Song-Qiang Xie, Dong Fang

**Affiliations:** 1 Institute of Chemical Biology, School of Pharmacy, Henan University, N. Jinming Ave, Kaifeng, 475004 China; 2 The Academy for Advanced Interdisplinary Studies, Henan University, N. Jinming Ave, Kaifeng, 475004 China

**Keywords:** Spermine, PD-L1, β-Catenin, STT3A, Hepatocellular carcinoma

## Abstract

**Background:**

Spermine is frequently elevated in tumor tissues and body fluids of cancer patients and is critical for cancer cell proliferation, migration and invasion. However, the immune functions of spermine in hepatocellular carcinoma progression remains unknown. In the present study, we aimed to elucidate immunosuppressive role of spermine in hepatocellular carcinoma and to explore the underlying mechanism.

**Methods:**

Whole-blood spermine concentration was measured using HPLC. Human primary HCC tissues were collected to examine the expression of CaSR, p-Akt, β-catenin, STT3A, PD-L1, and CD8. Mouse model of tumorigenesis and lung metastasis were established to evaluate the effects of spermine on hepatocellular carcinoma. Western blotting, immunofluorescence, real time PCR, digital Ca^2+^ imaging, and chromatin immunoprecipitation assay were used to investigate the underlying mechanisms by which spermine regulates PD-L1 expression and glycosylation in hepatocellular carcinoma cells.

**Results:**

Blood spermine concentration in the HCC patient group was significantly higher than that in the normal population group. Spermine could facilitate tumor progression through inducing PD-L1 expression and decreasing the CD8^+^ T cell infiltration in HCC. Mechanistically, spermine activates calcium-sensing receptor (CaSR) to trigger Ca^2+^ entry and thereby promote Akt-dependent β-catenin stabilization and nuclear translocation. Nuclear β-catenin induced by spermine then activates transcriptional expression of PD-L1 and *N*-glycosyltransferase STT3A, while STT3A in turn increases the stability of PD-L1 through inducing PD-L1 protein *N*-glycosylation in HCC cells.

**Conclusions:**

This study reveals the crucial function of spermine in establishing immune privilege by increasing the expression and *N*-glycosylation of PD-L1, providing a potential strategy for the treatment of hepatocellular carcinoma.

**Video Abstract**

**Supplementary Information:**

The online version contains supplementary material available at 10.1186/s12964-022-00981-6.

## Background

Spermine, a natural component from polyamine members, is essential for cellular growth, transformation and proliferation in eukaryotes [1, 2]. Cellular spermine level is tightly regulated by intricate regulation at multiple levels, i.e. biosynthesis, catabolism and transport [3, 4]. Interestingly, numerous studies have demonstrated that the enzymatic activities associated with polyamine biosynthesis are higher in cancer tissues than in normal surrounding tissues, resulting in an elevated level of spermine in tumor tissues and in body fluids (such as serum and urine) of cancer patients [5, 6]. Therefore, the level of spermine is regarded as one of the important cancer biomarkers and has been extensively studied to predict tumor progression [7–9].

The potential mechanisms of spermine-induced cancer cell growth and proliferation have also been investigated extensively [1]. It has been reported that spermine facilitate tumor progression through maintaining protein and nucleic acid synthesis, reducing reactive oxygen-induced damage, and regulating multiple ion channels necessary for cell-to-cell communication [10–12]. Depletion of polyamines including spermine usually causes growth arrest and apoptosis in cancer cells through the intrinsic mitochondrial pathway, as indicated by the changes in biochemical parameters, such as the loss of mitochondrial membrane potential, changes in the expression of Bcl-2 family proteins, release of cytochrome c and caspase 3 activation [13–15].

In addition to the direct and crucial roles in supporting cancer cell proliferation, spermine also plays a major role in regulating immune cell functions [16–18]. For example, spermine can inhibit M1 polarization, but promoted M2 polarization via upregulation of autophagy-related 5 (ATG5) expression in a mouse model of acute liver injury [19]; spermine could decrease the proliferation of natural killer cells and T lymphocytes in some autoimmune diseases [18, 20]; more importantly, spermine has been reported to suppress the sensitivity of cervical carcinoma cells to lymphokine-activated killer (LAK) lymphocytes [21]. Based on these studies, we supposed that spermine may act as an important immunosuppressive agent to facilitate cancer progression.

Hepatocellular carcinoma (HCC) is one of the most common cancers worldwide with high mortality and poor prognosis [22]. Accumulating evidence indicated that immune escape was one of the important contributors to the pathogenesis of HCC through remodeling the tumor microenvironment [23, 24]. Although spermine has been proven to be essential for HCC cell proliferation and viability [25], whether and how spermine contributes to tumor escape from immune surveillance in hepatocellular carcinoma was still unknown. In the present study, we provided evidence that spermine exerts an immunosuppressive role through enhancing the expression and glycosylation of programmed cell death ligand 1 (PD-L1, encoded by the CD274 gene) in hepatocellular carcinoma.

## Materials and methods

### Reagents and antibodies

Spermine (S4264), NPS2143 (SML0362), and BAPTA-AM (A1076) were purchased from Sigma-Aldrich (Saint Louis, MO, USA); MK2206 was purchased from Cell Signaling Technology (Beverly, MA, USA); N-Glycosidase F (P0704S) was obtained from New England BioLabs (Ipswich, MA, USA). Rabbit monoclonal anti-PD-L1 antibody (ab205921), rabbit monoclonal anti-CD8 alpha antibody (ab217344, ab237709), rabbit polyclonal anti-CaSR antibody (ab137408) were purchased from Abcam (Cambridge, MA, USA). Rabbit monoclonal anti-phospho-Akt (Ser473) (#4060), rabbit monoclonal anti-Akt (#4685), rabbit monoclonal anti-phospho-β-Catenin (Ser552) (#5651), rabbit monoclonal anti-β-Catenin (#8480) were purchased from Cell Signaling Technology (Beverly, MA, USA). Mouse monoclonal anti-STT3A (sc-100796) was obtained from Santa Cruz Biotechnology (Santa Cruz, CA); Rabbit polyclonal anti-STT3B (PA5-106380) was purchased from Thermo Fisher Scientific (Waltham, MA, USA). Mouse monoclonal anti-β-actin antibody (sc-47778), horseradish peroxidase (HRP)-conjugated goat anti-rabbit immunoglobulin G (IgG) (sc-2054) and goat anti-mouse IgG (sc-2973) were obtained from Santa Cruz Biotechnology (Santa Cruz, CA).

### Cell lines and cell culture

All cell lines used in the present study were purchased from the Cell Bank of the Chinese Academy of Science (Shanghai, China). These cells were authenticated by short tandem repeat Multi-amplification Kit (PowerPlex 16HS System). Cells were routinely cultured in Dulbecco’s modified Eagle’s medium supplemented with 10% fetal bovine serum (HyClone, Logan, UT, USA) at 37 °C in a humidified incubator containing 5% CO_2_.

### Cell transfections

PCR-amplified full-length human β-catenin, STT3A and STT3B cDNA were inserted into the pCMV-Flag-His-puro vector by Transheep (Shanghai, China), pCMV-Flag-His-β-catenin S552A was created using the QuikChange site-directed mutagenesis kit (Stratagene). pECE-myr-HA-Akt1 (delta4-129) were purchased from Addgene. For overexpression experiment, 2 μg plasmids were transfected into the indicated cancer cells with Lipofectamine 2000 reagent (Invitrogen, Thermo Fisher Scientific, Inc.) according to the manufacturer’s instructions. After 2 days, the cells were selected with 1.5 μg/ml puromycin (Sigma, St. Louis, USA) for 1 week and subjected to analyses.

The targeted shRNA sequences used to knock down expression of mouse PD-L1 was cloned into the pLKO.1-puro vectors. The target lentiviral constructs or control were transfected into HEK293T cells using PEI reagent (Polysciences, Warrington, USA). After 24 h transfection, the medium was replaced with fresh medium, and the lentivirus productions were subsequently collected and filtered at 72 h after transfection using 0.45 μm filters. Next, the lentivirus productions were added to the indicated cancer cells supplemented with 8 μg/ml polybrene (Millipore, Burlington, MA, USA). The transduced cells were selected in the presence of 1.5 μg/ml puromycin (Sigma, St. Louis, USA) for 1 week and subjected to analyses.

The small interfering RNA used to knockdown of β-catenin, STT3A, STT3B and control sequences were designed and synthesized Transheep (Shanghai, China). For transient transfection, the siRNA sequences were transfected with Lipofectamine 2000 (Invitrogen, Carlsbad, CA, USA) following the manufacturer’s instructions. After 24 h transfection, the cells were stimulated with 200 µM spermine for indicated time and subjected to analyses.

### Glycosylation analysis of PD-L1

To determine whether PD-L1 was glycosylated, cancer cells were lysed using the RIPA buffer containing 150 mM NaCl, 50 mM Tris (pH 7.5), 1% Nonidet P-40, and protease inhibitor mixture. The cell lysate was then denatured by heating at 100 °C for 10 min, followed by incubation with recombinant PNGase F (P0708S, New England Biolabs, MA, USA) at 37 °C for 1 h. Western blot analysis was performed to detect the effect of PNGase F on the molecular weight of PD-L1.

### Protein extraction and Western blotting

The total proteins were extracted from cancer cells using ice-chilled lysis buffer containing 50 mM tris–HCl (pH 8.0), 150 mM NaCl, 0.5% sodium deoxycholate, 0.1% SDS, 1% NP-40, 5 mM EDTA, 0.25 mM phenylmethylsulfonyl fluoride, and protease inhibitor cocktail. The nuclear proteins from cancer cells were isolated using ProteoExtract Subcellular Proteome Extraction Kit (Millipore). The concentration of protein was measured using a BCA assay kit (Pierce, Rockford, IL). Subsequently, a volume of 25–50 μg protein was separated by sodium dodecylsulfate-polyacrylamide gel electrophoresis (SDS-PAGE) gel and transferred onto a polyvinylidene difluoride membrane (Millipore, CA, USA). The membranes were then blocked for 1 h at room temperature with 5% skimmed milk or 3% bovine serum albumin dissolved in Tris-buffered saline with 0.1% Tween 20 (TBST) and incubated with the following primary antibodies at 4 ° overnight: Rabbit anti-PD-L1 (1:1000), rabbit anti-CaSR antibody (1:500), rabbit anti-phospho-Akt (Ser473) (1:1000), rabbit anti-Akt (1:1000), rabbit anti-phospho-β-catenin (Ser552) (1:500), rabbit anti-β-catenin (1:1000), mouse anti-STT3A (1:200), rabbit anti-STT3B (1:1000), mouse anti-β-actin (1:2000). After washed with TBST, the membranes were incubated with horseradish peroxidase-conjugated secondary antibodies (1:1000) for 1 h at room temperature. The blots were visualized with the enhanced chemiluminescence plus reagents and digitized into film images using a FluorChem E Imager System (Protein Simple, San Jose, CA, USA). Quantitation of intensity was performed from 8-bit linear TIF images by LabWorks software (Bio-Rad, USA).

### RNA isolation and real-time RT-PCR

Total RNA from cancer cells was isolated using TRIzol reagent (Invitrogen, CA, USA). Then 1 µg RNA was reverse‐transcribed to complementary DNA using PrimeScript RT‐polymerase (Takara, Shiga, Japan). Real-time PCR was performed using 2 × SYBR Green PCR Master Mix (Promega, WI, USA) on an ABI 7500 sequence detection system (Applied Biosystems, CA, USA). The amplification conditions were 5 min at 95 °C, then followed by 40 cycles of 15 s at 95 °C, 1 min at 60 °C. All PCR reactions were performed in triplicate. Glyceraldehyde-3-phosphate dehydrogenase (GAPDH) was used as an internal control for normalization. Relative quantification of the expression of genes was calculated using the 2^−(ΔΔCt)^ method.

The sequences of specific primers were as follows: human CD274, forward: 5′‐TGT CAG TGC TAC ACC AAG GC‐3′, reverse: 5′‐ACA GCT GAA TTG GTC ATC CC‐3′; human STT3A, forward: 5′‐GAA GCA ACA GGA TTC CAC CTA CC‐3′, reverse: 5′‐CAA TGG ACG GAG AAG AGT AGGC‐3′; human STT3B, forward: 5′‐GCA GGT GCT GTG TTC CTT AGT‐3′, reverse: 5′‐GTC GTA GGT TGA TGC TCA GAC AC‐3′; human GAPDH, forward: 5′‐GAC ACC CAC TCC TCC ACC TTT‐3′, reverse: 5′‐TTG CTG TAG CCA AAT TCG TTGT‐3′; mouse CD274, forward: 5′‐CTG CCA AAG GAC CAG CTT TT‐3′, reverse: 5′‐GGC TGG ATC CAC GGA AAT TC‐3′; mouse β-actin, forward: 5′‐TGG AAT CCT GTG GCA TCC ATG AAA‐3′, reverse: 5′‐TAA AAC GCA GCT CAG TAA CAG TCCG‐3′.

### Measurement of [Ca^2+^]cyt by digital Ca^2+^ imaging

SNU-368 cells cultured on coverslips were loaded with 5 μM Fura-2/AM (Invitrogen, NY, USA) in physiological salt solution (PSS) at room temperature for 1 h and then washed with PSS for 30 min. PSS used in digital Ca^2+^ measurement contained the following (in mM): 140 Na^+^, 5 K^+^, 2 Ca^2+^, 147 Cl^−^, 10 HEPES, and 10 glucose, pH 7.4. Next, cells on coverslips were placed and imaged in a standard perfusion chamber on the stage of an inverted fluorescence microscope (Nikon, Japan). Cells were treated with 200 μM spermine, and the ratio of Fura-2 fluorescence with excitation at 340 or 380 nm (F340/380) was followed over time and captured with an intensified CCD camera (ICCD200). To identify CaSR-mediating spermine-induced Ca^2+^ influx, a selective CaSR inhibitor NPS2134 (10 μM) were added to cells 5 min prior to spermine. Then, the baseline, peak, and amplitude of [Ca^2+^]_i_ responses were recorded and analyzed.

### Immunofluorescence

Cells were grown on coverslips and fixed for 10 min in 4% paraformaldehyde (w/v) at room temperature. After being permeabilized with 0.5% Triton X-100 for 15 min and blocked with 5% fetal calf serum for 1 h at room temperature, the cells were stained with rabbit monoclonal anti-β-catenin antibody (1:500) at 4 °C overnight. On the second day, the cells were washed three times with phosphate-buffered saline (PBS) and incubated with Alexa Fluor 488-conjugated secondary antibodies (1:1000, Invitrogen, CA, USA) for 1 h at room temperature. Nuclei was labeled with Hoechst 33,342 stain for 5 min at room temperature. Fluorescent images were captured using an LSM 510 M (Carl Zeiss) confocal microscope.

### Chromatin immunoprecipitation

Chromatin immunoprecipitation (ChIP) assays were performed using a SimpleChIP Plus Enzymatic Chromatin IP Kit (Cell Signaling Technology) in accordance with the instructions of the manufacturer. Briefly, cells were cross-linked with 1% formaldehyde for 10 min at 37 °C. Then, the chromatin was collected and enzymatically digested into fragments ranging from 150 to 900 base pair DNA/protein fragments. The DNA–protein complexes were immunoprecipitated using protein G-agarose beads and either 2 μg of the test ChIP-grade antibody. After that, protein-DNA cross-links were reversed by incubation at 65 °C overnight, and the DNA was purified by treatment with RNaseA, proteinase K, and multiple phenol: chloroform: isoamyl alcohol extraction. Real-time PCR were amplified using the following promoter primers: CD274, forward: 5′-ATG TAG CTC GGG ATG GGA AGT-3′, reverse: 5′-TGT GTG TGT GTG TAT GGG TGTA‐3′; STT3A, forward: 5′‐CAC ATG ACG GGA GTC AGTG‐3′, reverse: 5′‐CAA GCA GTT TGG TTG AGGG‐3′.

### Xenograft tumor assay

Male BALB/c mice aged 5 weeks were purchased from Beijing Weitong Lihua Animal Co. All mice were housed in an animal room with a 12-h light/dark cycle at a temperature of 20° to 24 °C and a relative humidity of 50 ± 10%. All animal procedures were conducted under the guidelines approved by the Institutional Animal Care and Use Committee at Henan University. Before the experimental procedures, all the mice were allowed 1 week of acclimatization. For the in vivo tumorigenesis experiment, the mice received a dorsal s.c. injection of 2 × 10^6^ H22 cells (in 100 μl PBS) with or without expression of PD-L1 shRNA. For tumor metastasis assays, 2 × 10^6^ H22 cells (in 100 μl PBS) were injected into the lateral tail vein of Balb/c mice. Then spermine (5 mg/kg) or equal volume of PBS was intraperitoneally administered into these mice once daily for two weeks from the day of tumor cell inoculation. After that, the mice were euthanized by cervical dislocation. Tumor tissues were excised and weighted before being fixed in 4% paraformaldehyde for IHC examinations. The lungs in the model of lung metastasis were removed and fixed with 4% paraformaldehyde for 1 day, the number of metastatic nodules on the lung surface was counted.

### Patients, tumor tissues and blood samples

Human primary HCC tissues and matched adjacent nontumour tissues were collected from 24 patients who were diagnosed and underwent surgical resection at the Huaihe Hospital of Henan University between 2019 and 2020. All tissue samples received no previous treatment for HCC before surgery. Blood samples were collected from 24 healthy volunteers and 24 cancer patients into tubes containing anticoagulant. Written informed consent was obtained from each patient or healthy volunteers at the time of tissue or blood sample collection. The study was approved by the Ethics Committee of Henan University.

### Immunohistochemistry (IHC)

The paraformaldehyde-fixed tumor tissues were dehydrated and embedded in paraffin. Then, the tissue specimens were cut into 4 µm pieces and placed on polylysine-coated slides, followed by xylene deparaffinization and ethanol gradient rehydration. To inactivate the activity of endogenous peroxidase, the slides were incubated with 3% H_2_O_2_ for 20 min at room temperature. After boiling in citrate buffer for antigen retrieval and cooling naturally, the slides were incubated overnight at 4 °C with the following antibodies: Rabbit anti-PD-L1 antibody (1:500), rabbit anti-CD8 alpha antibody (1:200), rabbit anti-CaSR antibody (1:500), rabbit anti-phospho-Akt (Ser473) (1:200), rabbit anti-β-Catenin (1:500), mouse anti-STT3A (1:500). Subsequently, the slides were incubated with streptavidin-conjugated HRP antibody for 30 min, and developed with diaminobenzidine.

Two independent pathologists analyzed the expression of the target protein using the semiquantitative method in a blinded manner, according to the percentage and intensity of positively stained cells. The percentage of positively stained cells was scored as follows: 0: if no tumor sections were stained; 1: if < 1% of sections were stained; 2: if 2–10% of sections were stained; 3: if 11–30% of sections were stained; 4: if 31–70% of sections were stained; or 5: if 71–100% of sections were stained. The staining intensity was scored on a scale of 0–3: 0 (negative), 1 (weak), 2 (moderate), or 3 (strong). The final IHC score of each tissue was generated by multiplying the percentage score with the staining score. The total number of intratumoral CD8^+^ T cells was counted in six low-power fields and presented as an average number of CD8^+^ T cells per low-power field.

### Determination spermine concentration in whole blood

The determination of whole-blood spermine concentration was performed as previously described [20]. Briefly, blood samples were vortexed and sonicated twice for 5 min before centrifugation at 18,000 g for 10 min. Then, the supernatants transferred to a new microtube, and an equal volume of 20% trichloroacetic acid, containing 20 µM N-(3-aminopropyl) cadaverine as an internal standard, was added. After centrifugation at 18,000 g for 10 min, the supernatant was separated and injected into a high performance liquid chromatography (HPLC) apparatus (Shimadzu, Kyoto, Japan) for analysis. Post-column derivatisation of eluted polyamines with o-phthalaldehyde yielded fluorescent products detectable at a wavelength of 450 nm after excitation at 345 nm.

### Statistical analysis

Statistical analysis was performed using Prism software 7.0 (GraphPad Software Inc., La Jolla, CA, USA). All data were presented as means ± SEM. The significance of the difference between two groups was evaluated using an unpaired Student’s t-test, while one-way analysis of variance (ANOVA) followed by Dunnett multiple comparison test or 2-way ANOVA followed by Bonferroni post hoc test was used to compare multiple groups. Spearman's rank correlation was used to examine the correlation of two proteins. A value of *p* < 0.05 was considered statistically significant.

## Results

### Spermine promoted HCC progression through inducing PD-L1 expression

**Fig. 1:**
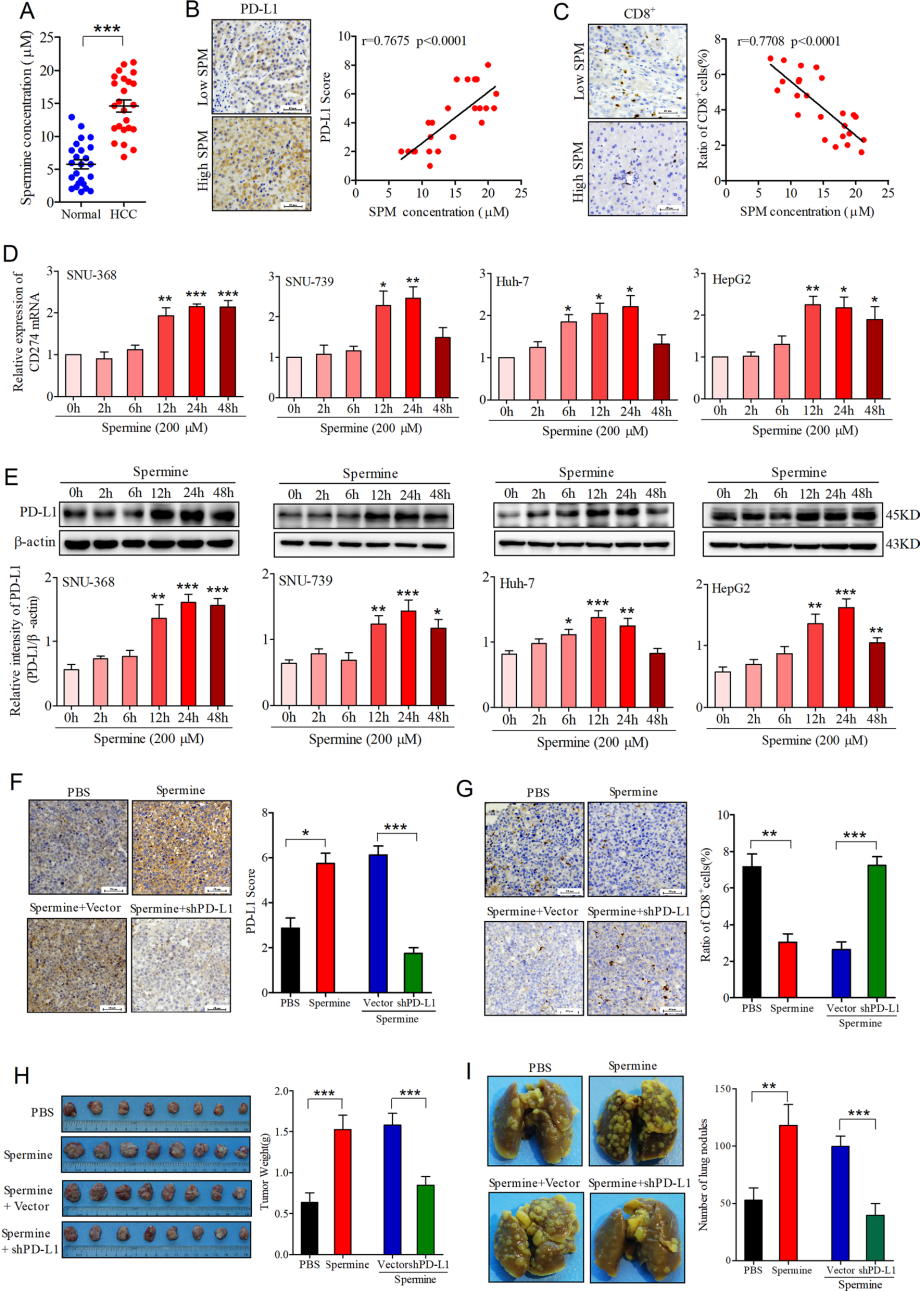
Spermine promotes tumor growth and metastasis through inducing PD-L1 expression in HCC. **A** Blood spermine was determined with HPLC in 24 normal subjects and 24 HCC patients. ***p < 0.001, two-tailed unpaired t test. **B** Spearman correlation test between blood spermine level and tumor PD-L1 IHC scores. Left: Representative images of IHC staining. Scale bar, 50 µm. Right: Correlation analysis. Note that some of the dots represent more than one specimen. **C** Spearman correlation test between blood spermine level and tumoral CD8^+^ T-cell infiltration. Left: Representative images of IHC staining from 24 human HCC specimens. Scale bar, 50 µm. Right: Correlation analysis. **D**, **E** Effects of spermine on the expression of CD274 mRNA (**D**) and PD-L1 protein (**E**) in HCC cells (SNU-368, SNU-739, Huh-7 and HepG2). *p < 0.05, **p < 0.01, ***p < 0.001 compared with control group, one-way ANOVA, n = 5 independent experiments per group. **F**, **G** IHC staining of tumoral PD-L1 (**F**) and CD8α (**G**) from tumor-grafted mice received with or without spermine treatment. Tumor-grafted mice was constructed with H22 tumor cells transfected with or without Ctrl shRNA or shPD-L1. Left: Representative images. Scale bar, 50 µm. Right: Correlation analysis. *p < 0.05, **p < 0.01, ***p < 0.001, one-way ANOVA, n = 8 mice per group. **H** Representative images of tumors and quantification of tumor weight collected from tumor-grafted mice received with or without spermine treatment. Tumor-grafted mice was constructed with H22 tumor cells transfected with or without Ctrl shRNA or shPD-L1. ***p < 0.001, one-way ANOVA, n = 8 mice per group. **I** Representative photos of metastatic lung nodules and quantification of the lung nodules from tumor-grafted mice received with or without spermine treatment. **p < 0.01, ***p < 0.001, one-way ANOVA, n = 8 mice per group

To assess the role of spermine in tumor immunity in HCC, we initiated our study by examining the blood spermine levels in HCC patients. HPLC analysis confirmed that blood spermine concentration in the HCC patient group was significantly higher than that in the normal population group (Fig. 1A). Meanwhile, IHC staining for PD-L1 and CD8 was conducted in tumors from the HCC patients. We found that the blood spermine levels were positively correlated with PD-L1 IHC score but negatively correlated with CD8^+^ T-cell infiltration in HCC tumors (Fig. 1B, C). To investigate whether spermine can stimulate the expression of PD-L1 in HCC cells, we then incubated four different HCC cells (SNU-368, SNU-739, Huh-7, and HepG2 cells) with 200 µM spermine and detected PD-L1 expression. The results revealed that spermine treatment significantly increased the CD274 mRNA and PD-L1 protein expression in these cancer cells (Fig. 1D, E).

Subsequently, we wondered whether spermine could promote PD-L1 expression in a tumorigenesis model of mice. To test it, we inoculated H22 mouse HCC cells subcutaneously into the left dorsal flank of BALB/c mice, then spermine (5 mg/kg) or equal volume of PBS was intraperitoneally administered into these mice once daily for two weeks from the day of tumor cell inoculation. Through IHC, we found an increased PD-L1 staining and a decreased CD8^+^ T cell population in the tumors from spermine-treated mice (Fig. 1F, G). However, when the tumor xenograft model of mice was established by inoculation of shPD-L1 cells, the decreased ratio of CD8^+^ T cells in tumors induced by spermine was significantly reversed (Fig. 1F, G). Through measuring tumor weight, we also demonstrated that spermine treatment can promote tumor growth, while knockdown of PD-L1 significantly attenuated spermine-induced tumor growth (Fig. 1H). Similarly, spermine treatment could significantly increase the numbers of tumor metastatic nodules in the model of lung metastasis, whereas knockdown of PD-L1 attenuated spermine-induced metastatic seeding in vivo (Fig. 1I). Collectively, these results indicated that spermine can promote tumor growth and metastasis by upregulating PD-L1 expression and decreasing the CD8^+^ T-cell infiltration.

### CaSR activation participated in spermine-induced PD-L1 expression in HCC

**Fig. 2:**
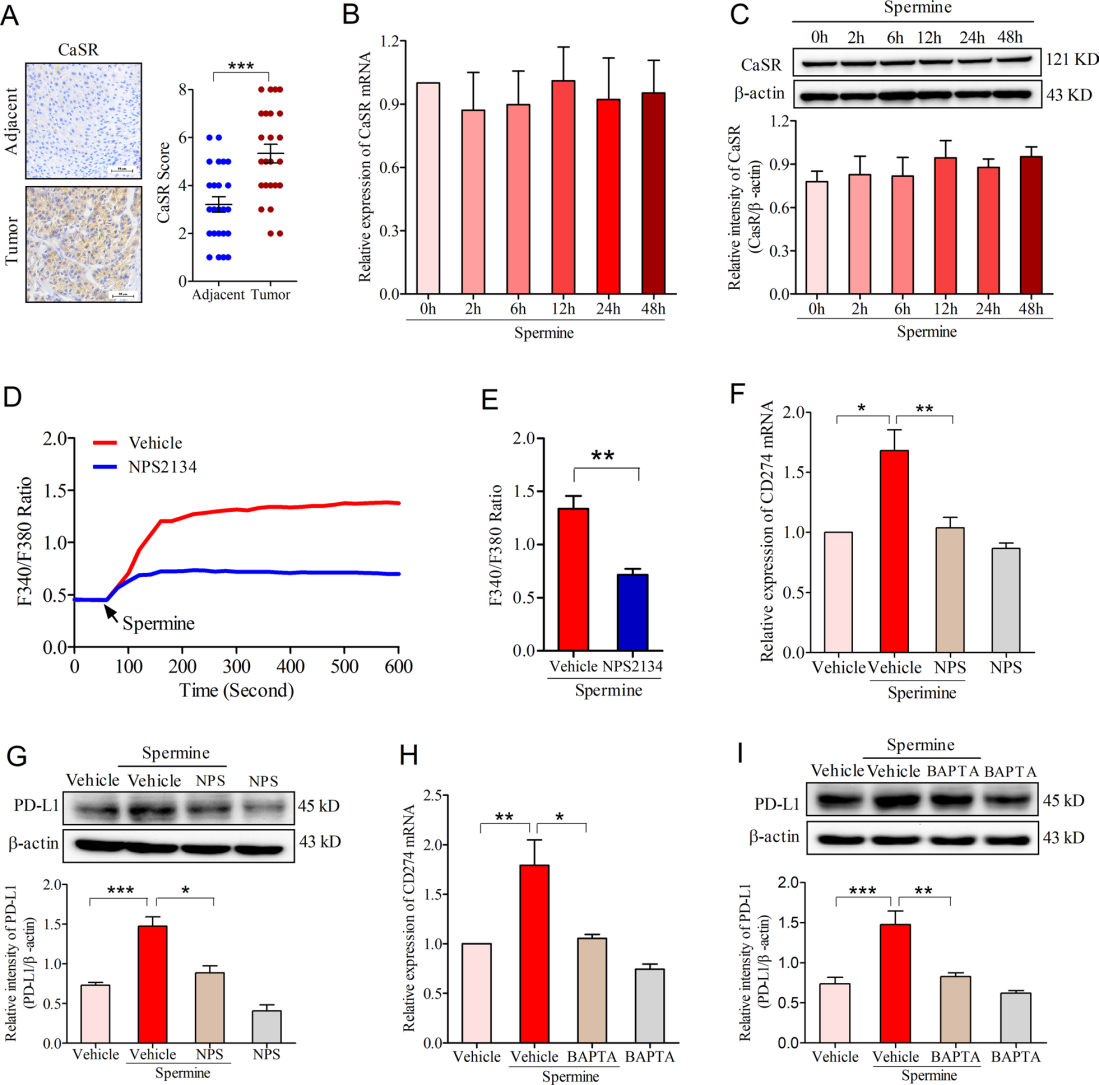
The activation of calcium-sensing receptor (CaSR) is required for spermine-induced PD-L1 expression in HCC. **A** Immunohistochemical staining of CaSR in the matched adjacent normal tissues and tumor tissues. ***p < 0.001, two-tailed unpaired t test, n = 24 samples per group. **B**, **C** Effects of spermine on the expression of CaSR Mrna (**B**) and protein (**C**) in SNU-368 cells. One-way ANOVA, n = 5 independent experiments per group. **D** Representative tracings of [Ca^2+^]_cyt_ changes in response to 200 µM spermine in the presence or the absence of NPS2134 (10 µM) in SUN368 cells. **I** Analysis of the changes in F340/F380 at 400 s after the stimulation with spermine in the presence or the absence of NPS2134. **p < 0.01, two-tailed unpaired t test, n = 4 independent experiments per group. **F**, **G** Effects of pretreatment with CaSR antagonist NPS2134 (10 µM; “NPS”) on the spermine-induced expression of CD274 Mrna (**F**) and PD-L1 protein (**G**) in SNU-368 cells. *p < 0.05, **p < 0.01, ***p < 0.001, one-way ANOVA, n = 5 independent experiments per group. **H**, **I** Effects of pretreatment with Ca^2+^ chelator BAPTA-AM (10 µM; “BAPTA”) on the spermine-induced expression of CD274 mRNA (**H**) and PD-L1 protein (**I**) in SNU-368 cells. *p < 0.05, **p < 0.01, ***p < 0.001, one-way ANOVA, n = 4 independent experiments per group

Accumulating evidence have suggested that spermine performed its biological function through binding to calcium-sensing receptor (CaSR), a member of the G protein-coupled receptor (GPCR) superfamily [26–28]. Therefore, we first examined the expression of CaSR in human HCC and adjacent normal tissues. IHC analyses showed that CaSR expression was enhanced in the tumor tissues (Fig. 2A). Then, we asked whether spermine could affect CaSR expression in HCC cells. Through real time PCR and Western blot, we observed that spermine did not affect the mRNA and protein expression of CaSR in SNU-368 cells (Fig. 2B, C).

CaSR has been reported to act through G proteins to trigger cytosolic Ca^2+^ increase after being activated by spermine [29]. Consistent with these reports, we also found that spermine (200 µM) treatment significantly evokes an increase in intracellular Ca^2+^ concentration in SNU-368 cells, and a CaSR inhibitor NPS2134 (10 µM) could block spermine-induced Ca^2+^ influx (Fig. 2D, E). To investigate whether the activation of CaSR by spermine is required for PD-L1 expression, we examined the effects of the CaSR inhibitor NPS2134 and the intracellular Ca^2+^ chelator BAPTA-AM (each applied to cells at 10 µM) on spermine-induced expression of CD274 mRNA and PD-L1 protein in HCC cells. As shown, both NPS2134 and BAPTA-AM abrogated spermine-induced upregulation of CD274 mRNA and PD-L1 protein expression in SNU-368 cells (Fig. 2F–I).

### Akt activation was instrumental for spermine-induced PD-L1 expression

**Fig. 3:**
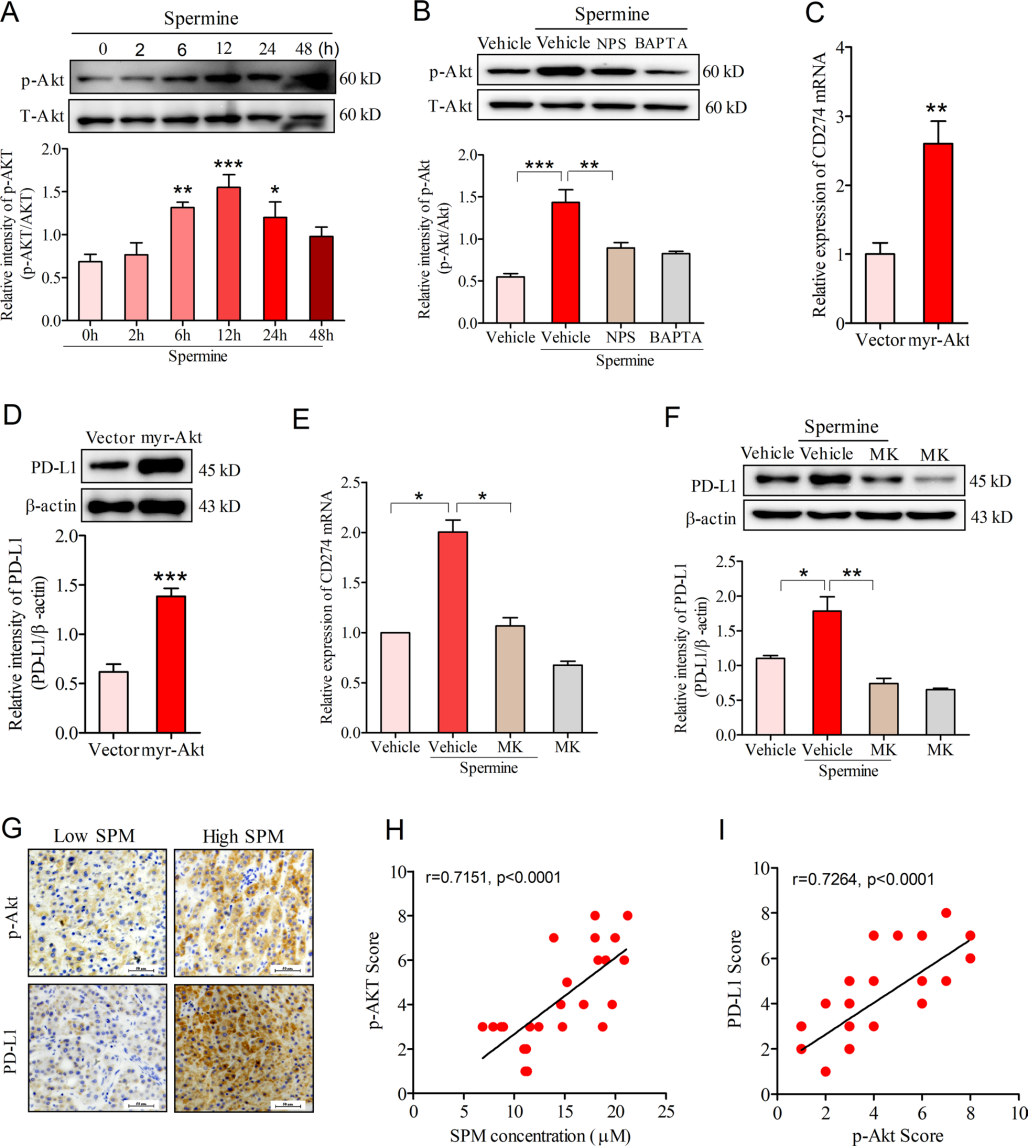
Akt activation is involved in spermine-mediated PD-L1 expression in HCC. **A** Effects of spermine (200 µM) on the level of Akt Ser473 phosphorylation in SNU-368 cells. *p < 0.05, **p < 0.01, ***p < 0.001, one-way ANOVA, n = 5 independent experiments per group. **B** Effects of pretreatment with 10 µM NPS2134 or 10 µM BAPTA-AM on spermine-induced increased phosphorylation levels of Akt at Ser473 in SNU-368 cells after 12 h of coincubation. **p < 0.01, ***p < 0.001, one-way ANOVA, n = 5 independent experiments per group. **C**, **D** Effects of ectopic expression of myr-Akt1 isoform on endogenous CD274 mRNA (**C**) and PD-L1 protein (**D**) in SNU-368 cells. **p < 0.01, ***p < 0.001, two-tailed unpaired t test, n = 5 independent experiments per group. **E**, **F** Effects of pretreatment with Akt inhibitor MK2206 (10 µM, “MK”) on spermine-induced the expression of CD274 mRNA (**E**) and PD-L1 protein (**F**) in SNU-368 cells after incubation with spermine for 24 h. **p < 0.01, ***p < 0.001, one-way ANOVA, n = 5 independent experiments per group. **G** Immunohistochemical staining of phosphor-Akt and PD-L1 in 24 human HCC specimens. Scale bar, 50 μm. **H** Spearman correlation test between blood spermine concentration and tumor phosphor-Akt IHC scores. **I** Spearman correlation analysis of phosphor-Akt and PD-L1. Note that the scores of some samples overlap

Akt was reported to be an immediate responder to CaSR activation in gastric cancer cells [30]. Therefore, we explored whether elevated [Ca^2+^]i upon spermine stimulation triggers the activation of Akt signaling pathway in HCC cells. After incubation with 200 µM spermine for 6–24 h, the phosphorylation of Akt at Ser473 was enhanced in SNU-368 (Fig. 3A). Pretreatment of SNU-368 cells with CaSR inhibitor NPS2134 or the fast Ca^2+^ chelator BAPTA-AM (each applied to cells at 10 µM) significantly attenuated the phosphorylation of Akt Ser473 in these cells (Fig. 3B).

To test whether Akt activation was involved in spermine-induced PD-L1 expression, we then expressed constitutively active, myristoylated Akt1 (myr-Akt1) in SNU-368 cells and examined PD-L1 expression. The results showed that expression of myr-Akt1 significantly enhanced CD274 mRNA and PD-L1 protein expression levels in HCC cells (Fig. 3C, D). In contrast, treatment with Akt inhibitor MK2206 could inhibit spermine-induced CD274 mRNA and PD-L1 protein expression levels in SNU-368 cells (Fig. 3E, F). Subsequently, we performed IHC staining to examine the expression of p-Akt and PD-L1 in HCC tumor tissues (Fig. 3G). Through Spearman rank correlation test, we found that blood spermine was positively correlated with the p-Akt expression level in tumors (Fig. 3H). Moreover, positive correlation was also detected between p-Akt and PD-L1 IHC scores in the human HCC tumors (Fig. 3I). Together, these results indicated that spermine could promote PD-L1 expression through the activation of Akt in HCC.

### Akt-dependent β-catenin phosphorylation and nuclear accumulation mediated spermine-induced PD-L1 expression

**Fig. 4:**
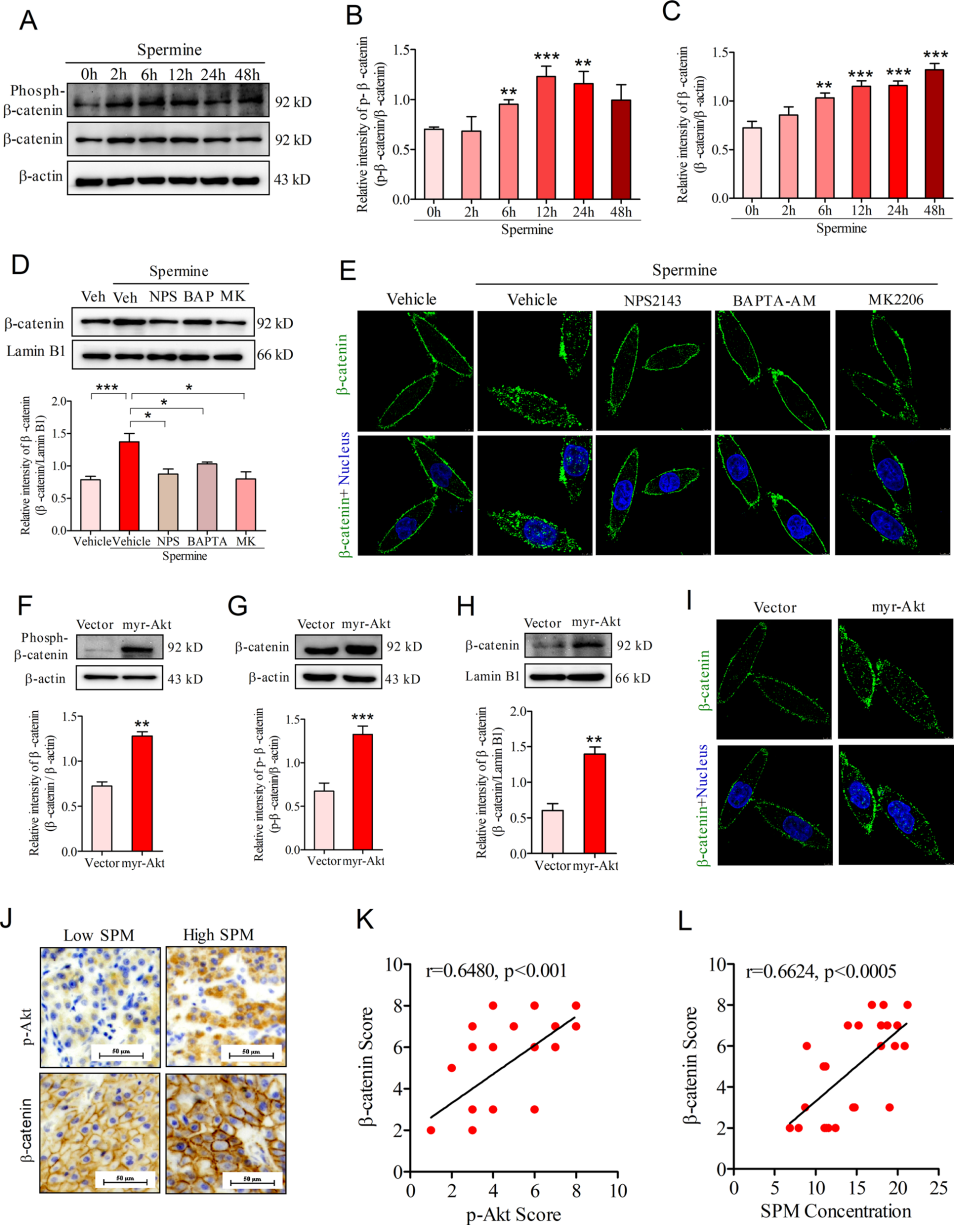
Spermine promotes β-catenin phosphorylation and nuclear accumulation through the activation of Akt in HCC. **A** Western blot of the phosphorylation level and total protein expression of β-catenin in SNU-368 cells incubated with spermine. **B**, **C** Analysis of effects of spermine on the phosphorylation level (**B**) and total protein (**C**) expression of β-catenin. **p < 0.01, ***p < 0.001, one-way ANOVA, n = 5 independent experiments per group. **D** Effects of pretreatment with NPS2134 (10 µM), BAPTA-AM (10 µM), MK2206 (10 µM) on the spermine-induced β-catenin nuclear accumulation in SNU-368 cells after incubation with spermine for 12 h. Veh: Vehicle; NPS: NPS2134; BAP: BAPTA-AM; MK: MK2206. *p < 0.05, ***p < 0.001, one-way ANOVA, n = 5 independent experiments per group. **I** Immunofluorescence staining of β-catenin in SNU-368 cells. The cells were pretreated with NPS2134 (10 µM), BAPTA-AM (10 µM), MK2206 (10 µM) for 30 min before coincubation with spermine for 12 h. n = 3 independent experiments per group. **F**, **G** Effects of ectopic expression of myr-Akt1 isoform on the phosphorylation level (**F**) and total protein (**G**) expression of β-catenin in SNU-368 cells. **p < 0.01, ***p < 0.001, two-tailed unpaired t test, n = 5 independent experiments per group. **H** Effects of ectopic expression of myr-Akt1 isoform on β-catenin nuclear accumulation in SNU-368 cells. **p < 0.01, two-tailed unpaired t test, n = 4 independent experiments per group. **I** Immunofluorescence staining of β-catenin in SNU-368 cells stably expressing myr-Akt1. n = 3 independent experiments per group. **J** IHC staining of phosphor-Akt and β-catenin in 24 human HCC specimens. Scale bar, 50 μm. **K** Spearman correlation analysis of phosphor-Akt and β-catenin IHC scores. Note that the scores of some samples overlap. **L** Spearman correlation test between blood spermine concentration and tumor β-catenin IHC scores

Considering that Akt-mediated β-catenin S552 phosphorylation and nuclear β-catenin enhanced PD-L1 expression in human glioblastoma [31], we wondered whether Akt-dependent phosphorylation and nuclear accumulation of β-catenin participated in spermine-mediated PD-L1 expression in HCC. After treatment with spermine, both the phosphorylation level and total protein expression of β-catenin was enhanced in SNU-368 cells (Fig. 4A–C). Moreover, we also observed that spermine could promote nuclear accumulation of β-catenin in SNU-368 cells, while CaSR inhibitor NPS2134, the fast Ca^2+^ chelator BAPTA-AM and Akt inhibitor MK2206 significantly blocked spermine-induced nuclear β-catenin expression in SNU-368 cells (Fig. 4D). Immunofluorescence analysis further confirmed these results (Fig. 4E).

Subsequently, we transfected SNU-368 cells with myr-Akt1 and examined β-catenin expression. The results showed that introduction of myr-Akt1 significantly increased β-catenin phosphorylation and total protein expression in SNU-368 cells (Fig. 4F, G). Meanwhile, reconstituted expression of myr-Akt1 also induced β-catenin nuclear accumulation in these cells (Fig. 4H, I). In support of these in vitro results, we observed a positive correlation between p-Akt and β-catenin IHC scores in HCC tumor tissues (Fig. 4J, K). In addition, our results also indicated that HCC patients with higher blood spermine showed a higher β-catenin IHC score in tumor tissues (Fig. 4L). Obviously, these results suggested that Akt activation is instrumental for spermine-induced β-catenin phosphorylation and nuclear accumulation.

**Fig. 5:**
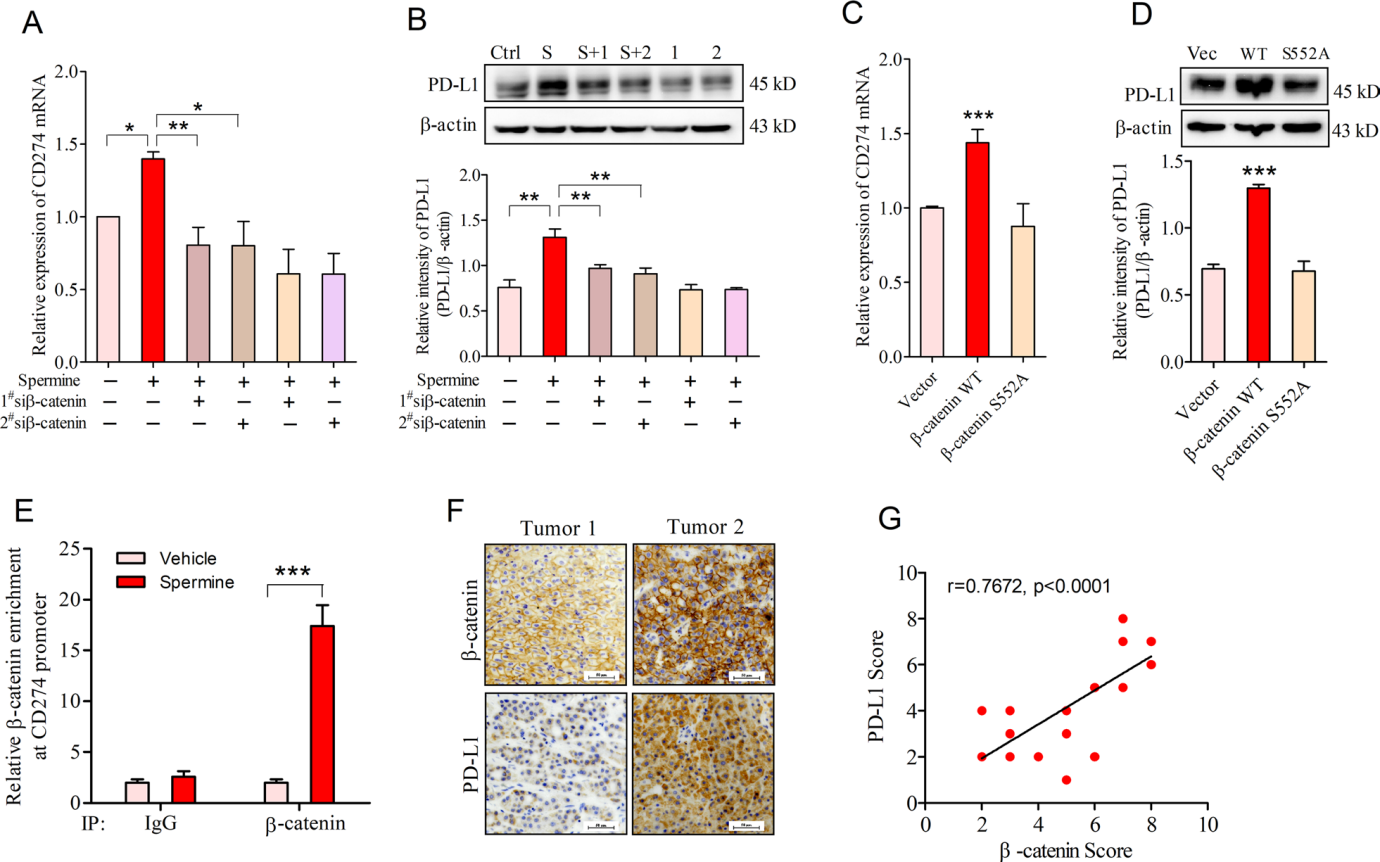
Akt-dependent β-catenin phosphorylation mediated spermine-induced PD-L1 expression. **A**, **B** Effects of β-catenin knockdown on spermine-induced CD274 mRNA (**A**) and PD-L1 protein (**B**) expression in SNU-368 cells. S: spermine; 1: 1^#^β-catenin siRNA; 2: 2^#^ β-catenin siRNA. *p < 0.05, **p < 0.01, one-way ANOVA, n = 5 independent experiments per group. **C**, **D** The expression of CD274 mRNA (**C**) and PD-L1 protein (**D**) in SNU-368 cells expressing WT β-catenin or β-catenin S552A mutant for 24 h. ***p < 0.001 compared with vector group, one-way ANOVA, n = 5 independent experiments per group. **E** ChIP analysis of the amounts of β-catenin recruited to the CD274 promoter regions in SNU-368 cells treated with spermine (200 µM) for 24 h. ***p < 0.001, two-tailed unpaired t test, n = 4 independent experiments per group. **F** IHC staining of β-catenin and PD-L1 in 24 human HCC specimens. Scale bar, 50 μm. **G** Spearman correlation analysis of β-catenin and PD-L1 IHC scores. Note that the scores of some samples overlap

To identify Akt-dependent phosphorylation and nuclear accumulation of β-catenin was involved in spermine-mediated PD-L1 expression in HCC, we incubated β-catenin-knockdown SNU-368 cells with spermine and examined PD-L1 expression. The results showed that knockdown of β-catenin using two specific siRNA significantly suppressed spermine-induced the upregulation of CD274 mRNA and PD-L1 protein expression (Fig. 5A, B). Then, we transfected WT β-catenin or β-catenin S552A mutant into SNU-368 cells and examined PD-L1 expression. As the results shown, overexpression of WT β-catenin, but not the β-catenin S552A mutant, which is resistant to phosphorylation and activation by Akt, largely increased CD274 mRNA and PD-L1 protein expression in SNU-368 cells (Fig. 5C, D). To further confirm that β-catenin was involved in spermine-induced transcriptional expression of PD-L1, we performed ChIP assay with an anti-β-catenin antibody. The results showed that treatment of SNU-368 cells with spermine increased the binding of β-catenin to the promoter region of CD274 (Fig. 5E). Meanwhile, IHC staining for β-catenin and PD-L1 was also conducted in HCC tumor tissues, correlation analysis revealed that β-catenin expression is positively correlated with levels of PD-L1 in a statistically significant manner (Fig. 5F, G).

### STT3A mediated PD-L1 N-glycosylation in response to spermine stimulation

**Fig. 6:**
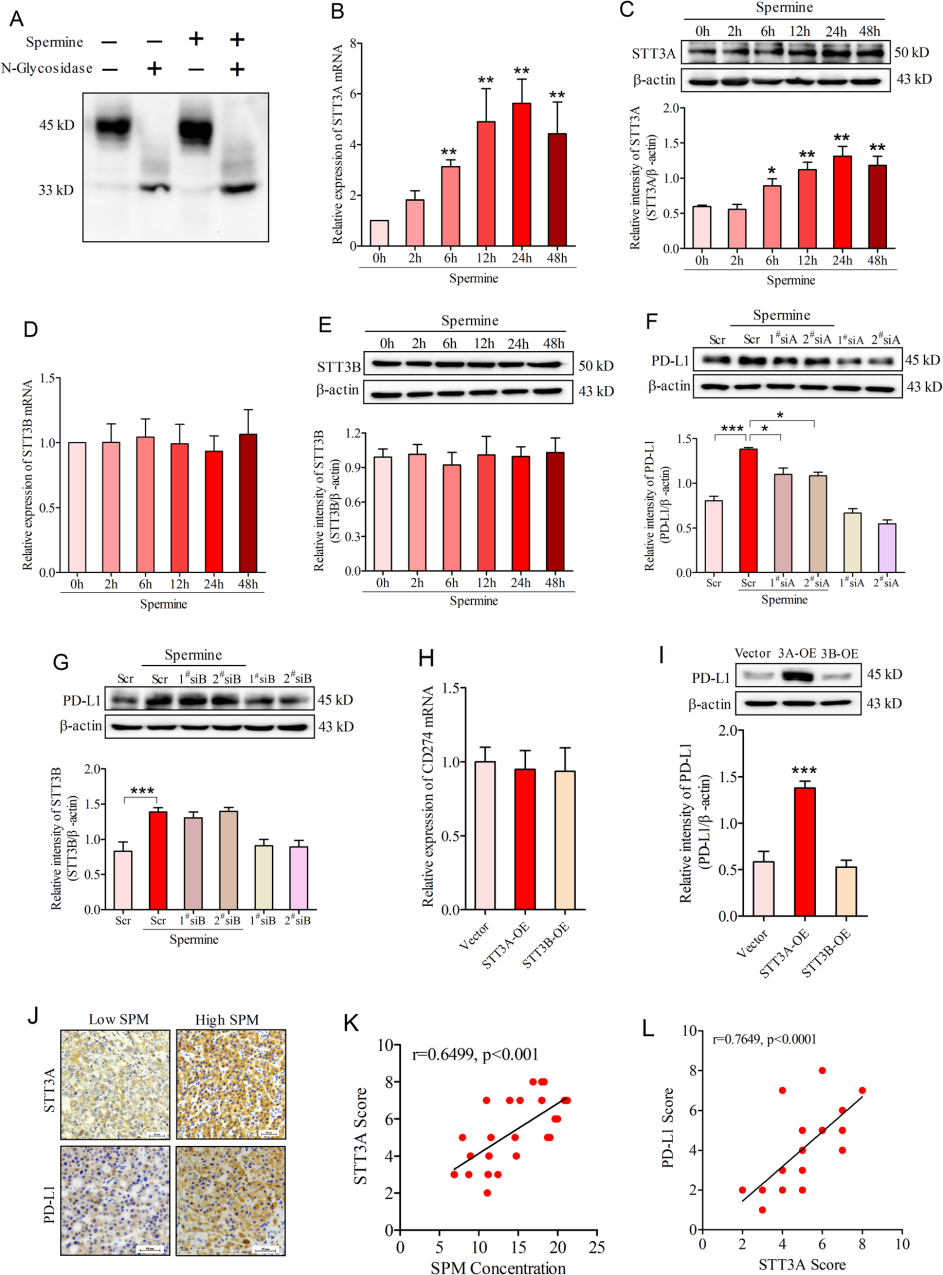
Spermine promotes PD-L1 protein N-glycosylation and stability through inducing STT3A expression in HCC. **A** Effects of PNGase F on the molecular weight of PD-L1 in the lysates of SNU-368 cells treated with or without spermine (200 µM) for 24 h. **B**, **C** Effects of spermine (200 µM) on the expression of STT3A mRNA (**B**) and protein (**C**) in SNU-368 cells. *p < 0.05, **p < 0.01, one-way ANOVA, n = 5 independent experiments per group. **D**, **E** Effects of spermine (200 µM) on the expression of STT3B mRNA (**D**) and protein (**E**) in SNU-368 cells. one-way ANOVA, n = 5 independent experiments per group. **F**, **G** Effects of STT3A (**F**) and STT3B (**G**) knockdown on spermine-induced PD-L1 protein expression in SNU-368 cells. 24 h after two specific siRNA transfection, cells were incubated in the presence or absence of spermine (200 µM) for 24 h. Scr: Scramble; 1^#^siA: 1^#^ STT3A siRNA; 2^#^siA: 2^#^ STT3A siRNA; 1^#^siB: 1^#^ STT3B siRNA; 2^#^siB: 2^#^ STT3B siRNA; *p < 0.05, ***p < 0.001, one-way ANOVA, n = 5 independent experiments per group. **H**, **I** The expression of CD274 mRNA (**H**) and PD-L1 protein (**I**) in SNU-368 cells expressing STT3A or STT3B for 24 h. ***p < 0.001 compared with Vector group, one-way ANOVA, n = 4 independent experiments per group. **J** IHC staining of STT3A and PD-L1 in 24 human HCC specimens. Scale bar, 50 μm. **K** Spearman correlation test between blood spermine concentration and tumor STT3A IHC scores. **L** Spearman correlation analysis of STT3A and PD-L1 IHC scores. Note that the scores of some samples overlap

β-Catenin reportedly transcriptionally activates N-glycosyltransferase STT3 isoforms, which in turn regulates PD-L1 induction through PD-L1 protein N-glycosylation and stability [32]. Given that the molecular weight of PD-L1 in the present study was around 45 kDa, a molecular weight was closed to that of N-glycosylated PD-L1, we asked whether spermine-induced nuclear localization of β-catenin transcriptionally upregulates STT3 isoforms, leading to PD-L1 glycosylation and stabilization in HCC. To answer this question, we first incubated HCC cell lysates with recombinant N-glycosidase F (PNGase F) and detected the changes of PD-L1. Western blot analysis showed that PNGase F treatment caused a molecular weight shift of PD-L1 from 45 to 33 kDa in the cell lysates extracted from SNU-368 cells incubated with or without spermine (Fig. 6A). These findings suggested that PD-L1 was also subjected to protein N-glycosylation.

To explore whether the N-glycosyltransferase STT3 was responsible for PD-L1 N-glycosylation upon spermine stimulation, we firstly treated HCC cells with spermine and analyzed the expression of STT3 isoforms (including A and B two isoforms in mammalian cells). The results showed that spermine treatment could upregulate STT3A mRNA and protein expression in SNU-368 cells (Fig. 6B, C), while STT3B expression at both mRNA and protein levels remained unchanged in response to spermine stimulation (Fig. 6D, E). Then we knocked down endogenous STT3 isoforms in hepatocellular carcinoma cells and found that depletion of STT3A, but not STT3B, significantly suppressed spermine-mediated PD-L1 induction in protein levels (Fig. 6F, G). To further confirm the roles of STT3A and STT3B in PD-L1 induction, we transfected STT3A-overexpression plasmids into SNU-368 cells and examined the expression levels of CD274 mRNA and PD-L1 protein. Results showed that ectopic expression of STT3A or STT3B did not significantly affect CD274 mRNA expression, but overexpression of STT3A could induce PD-L1 protein expression (Fig. 6H, I). In support of these in vitro results, we also demonstrated that STT3A expression was positively correlated with blood spermine and tumoral PD-L1 expression in HCC patients (Fig. 6J, L).

**Fig. 7:**
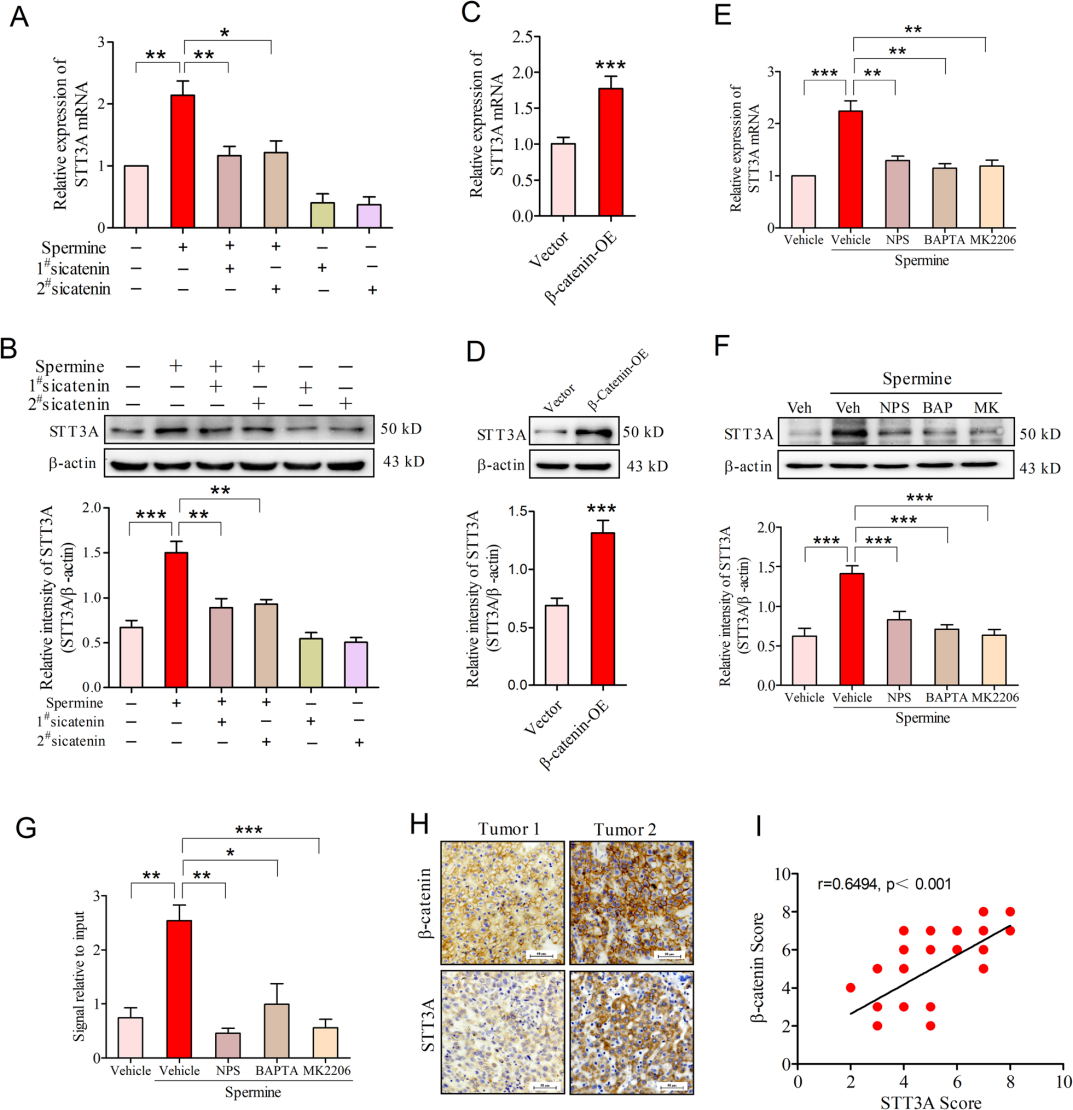
β-Catenin was involved in spermine-induced STT3A expression at transcriptional level. **A**, **B** Effects of β-catenin knockdown on spermine-induced STT3A mRNA (**A**) and protein (**B**) expression in SNU-368 cells. 24 h after two specific siRNA transfection, cells were incubated in the presence or absence of spermine (200 µM) for 24 h. *p < 0.05, **p < 0.01, ***p < 0.001, one-way ANOVA, n = 5 independent experiments per group. **C**, **D** The expression of STT3A mRNA (**C**) and protein (**D**) in SNU-368 cells expressing WT β-catenin for 24 h. ***p < 0.001, two-tailed unpaired t test, n = 4 independent experiments per group. **E**, **F** Effects of pretreatment with NPS2134 (10 µM), BAPTA-AM (10 µM), MK2206 (10 µM) on the spermine-induced STT3A mRNA I and protein (**F**) expression in SNU-368 cells after incubation with spermine for 24 h. Veh: Vehicle; NPS: NPS2134; BAP: BAPTA-AM; MK: MK2206. **p < 0.01, ***p < 0.001, one-way ANOVA, n = 5 independent experiments per group. **G** Effects of pretreatment with NPS2134 (10 µM), BAPTA-AM (10 µM), MK2206 (10 µM) on the amounts of β-catenin recruited to the STT3 promoter regions in SNU-368 cells treated with spermine (200 µM) for 24 h. *p < 0.05, **p < 0.01, ***p < 0.001, one-way ANOVA, n = 4 independent experiments per group. **H** IHC staining of β-catenin and STT3A in 24 human HCC specimens. Scale bar, 50 μm. **I** Spearman correlation analysis of β-catenin and STT3A IHC scores. Note that the scores of some samples overlap

Subsequently, we sought to determine whether β-catenin was involved in spermine-induced STT3A expression at transcriptional level. As shown, knockdown of β-catenin significantly abrogated spermine-induced up-regulation of STT3A mRNA and protein expression in SNU-368 cells (Fig. 7A, B). In contrast, introduction of exogenous β-catenin was sufficient to induce STT3A expression at both mRNA and protein levels in these cancer cells (Fig. 7C, D). Then, we used the CaSR inhibitor NPS2134, the fast Ca^2+^ chelator BAPTA-AM and Akt inhibitor MK2206 to block spermine-induced β-catenin nuclear accumulation and examined STT3A expression in HCC cells. As expected, all of these inhibitors could reverse spermine-induced the upregulation of STT3A mRNA and protein expression in SNU-368 cells (Fig. 7E, F). ChIP assays also suggested that spermine treatment increased the binding of β-catenin to the promoter region of STT3A, while NPS2134, BAPTA-AM and MK2206 could reduce the binding of β-catenin to the promoter region of STT3A in SNU-368 cells (Fig. 7G). In addition, we also observed that β-catenin expression was positively correlated with STT3A expression in human tumors (Fig. 7H, I). Collectively, these results indicated that β-catenin was instrumental for spermine-induced the up-regulation of STT3A at transcriptional level in HCC.

## Discussion

**Fig. 8:**
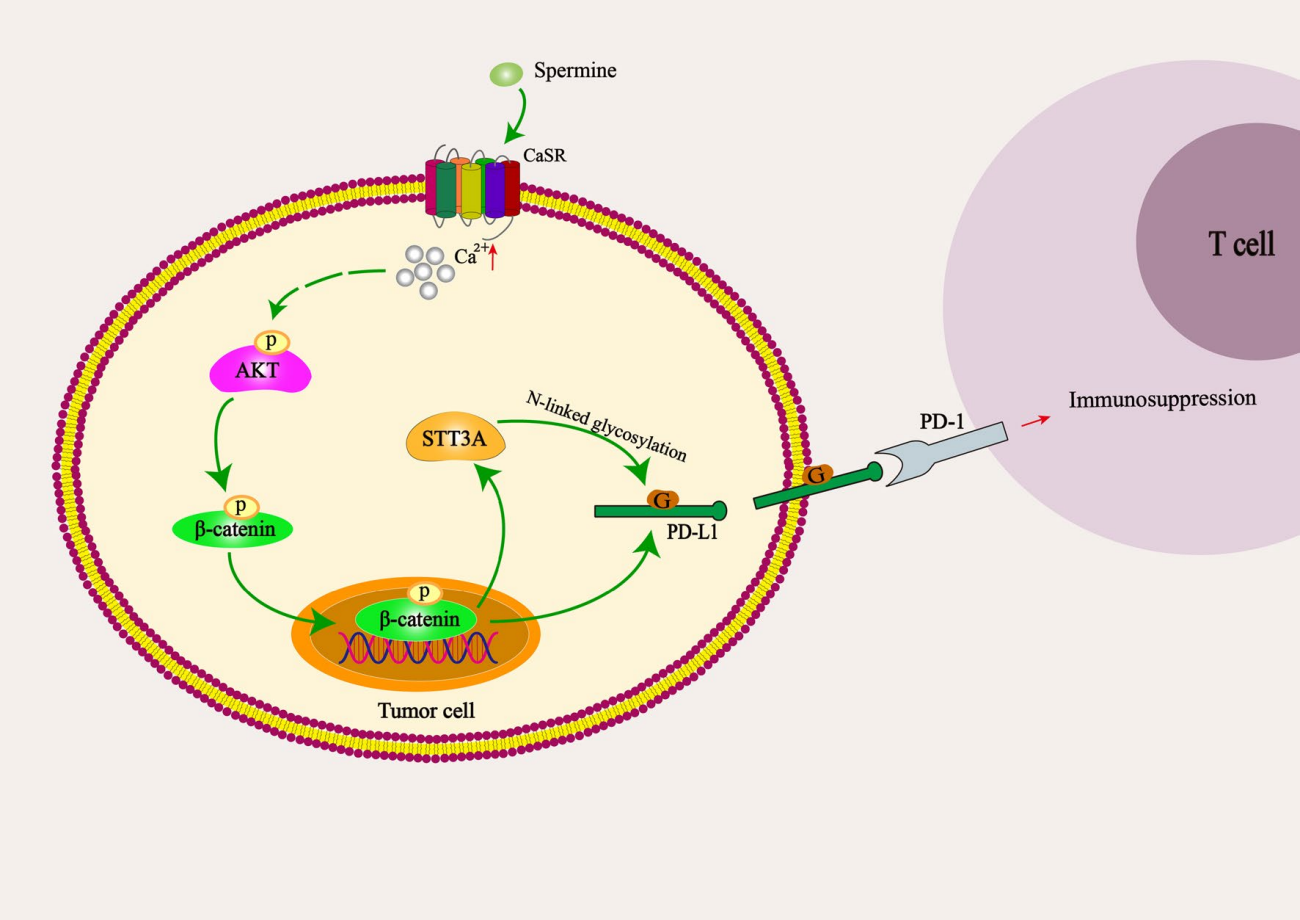
Schematic representation of spermine-mediated PD-L1 expression and glycosylation in HCC. Spermine triggers Akt-dependent β-catenin phosphorylation at Ser552 through acting on CaSR, phosphorylated β-catenin then translocates into the nucleus where it transcriptionally activates PD-L1 and STT3A expression by binding to their promoter regions. On the other hand, STT3A stabilizes PD-L1 by modulating protein N-glycosylation, resulting in the suppression of cytotoxic T cell activity in the tumor microenvironment

In addition to the direct roles of supporting cell growth, transformation and proliferation in eukaryotes, spermine reportedly acted as an inhibitor of immune responses under some pathological conditions [20, 21]. Here, our study first revealed that spermine could suppress cytotoxic T cell activity in the tumor microenvironment through enhancing the expression and N-glycosylation of PD-L1 in hepatocellular carcinoma (Fig. 8). Notably, in most of our in vitro tests, spermine-induced the activation of Akt-β-catenine-STT3A pathway and elevation of PD-L1 expression only lasted 24 h and recovered after 48 h. It may be because spermine is consumed or decomposed after 24 h in vitro. Considering that spermine is continuously synthesized in the tumor cells of cancer patients [6], we suppose that spermine-induced the modification of PD-L1 plays a critical role in remodeling immunosuppressive microenvironment in HCC.

CaSR, a member of G protein-coupled receptor (GPCR) subfamily C, is widely distributed in many organs and tissues such as brain, lung, gastrointestinal tract, and smooth muscle [26, 33, 34]. The ligands or activators of CaSR include polyvalent cations (e.g., Ca^2+^, Mg^2+^, Gd^3+^) and polycationic molecules like spermine [35, 36]. Besides sensing extracellular calcium and signaling to maintain Ca^2+^ homeostasis, CaSR influences a wide variety of cellular processes that are involved in inflammation, the nervous and cardiovascular system [37–39]. What is more important, CaSR has been reported to play a critical role in either promoting or suppressing the progression of several human cancers [35, 40]. For example, CaSR overexpression in breast, lung, prostate and kidney cancers were positively correlated with tumor progression and shorter survival; inhibition or silence of CaSR markedly suppressed cancer cell proliferation, invasion, and migration [26, 41]. Mechanistic studies have suggested that CaSR promotes tumor growth and metastasis through regulating multiply signaling pathways, such as PLC-PKC, MEK-ERK and PI3K-Akt pathways [42, 43]. Notably, Xie et al. found that activation of CaSR by spermine could promote cell growth and migration through a Ca^2+^/Akt/β-catenin relay in gastric cancer cells [30]. In line with their report, we also demonstrated that spermine stimulation activates CaSR, which in turn mediates Ca^2+^ entry to increase [Ca^2+^]cyt and consequent activation of Akt and β-catenin in hepatocellular carcinoma cells. In contrast, reduced expression of CaSR has been observed in colorectal, pancreatic and neuroblastomas cancers, which was associated with an increase in the progression of malignancy [44–46]. Especially, a study also revealed that CaSR acts as a suppressor in pancreatic tumorigenesis through inhibition of β-catenin activity [45]. These discrepancies of the roles of CaSR on tumor progression in different cancers imply that it might be tissue or disease specific. Interestingly, CaSR is not only expressed on tumor cells, but also exists in T lymphocytes [47, 48]. It was found that the activation of CaSR in T lymphocytes induces the secretion of cytokines and T-cell apoptosis [49]. Therefore, we speculate that spermine may directly activate CaSR in T lymphocytes and induce the apoptosis of T lymphocytes, leading to immune escape of tumor cells in HCC.

As a key component of the canonical Wnt signaling pathway, β-catenin has been implicated in the tumor initiation, growth and metastasis of various cancers [50]. Numerous studies suggested that β-catenin expression could be tightly regulated by the PI3K/Akt signaling pathway [51, 52]. For example, Akt is capable of phosphorylating glycogen synthase kinase-3β (GSK-3β) at Ser9 residue, which leads to the inactivation of GSK-3β in the β-catenin-destruction complex, and thus, results in β-catenin accumulation and nuclear translocation [53, 54]. On the other hand, Akt could directly phosphorylate β-catenin at Ser552 residue, thereby contributing to β-catenin stability and nuclear translocation. In the present study, we demonstrated spermine could phosphorylate β-catenin at Ser552 residue through activation of Akt in HCC cells [31, 55]. Of course, we did not exclude that spermine promoted β-catenin accumulation and nuclear translocation through inactivation of GSK-3β, because spermine could inactivates GSK-3β through PI3K-Akt-GSK-3β pathway [56]. Consistent with earlier findings, we also found that phosphorylation of β-catenin at Ser 552 by Akt contributes to β-catenin stability, transcriptional activity, and increase STT3 and PD-L1 expression. Interestingly, a recent study disclosed that PD-L1 can upregulate β-catenin by inhibiting its degradation through PI3K/Akt signaling pathway [57]. Taken together with our results, it raises the possibility that β-catenin and PD-L1 may establish a positive feedback loop and accelerate tumor progression.

The regulatory mechanisms of PD-L1 expression could be occurred at several levels including the level of transcription, post-transcription and post-translation [58, 59]. Interestingly, spermine not only promoted PD-L1 expression at transcriptional level, but also induced PD-L1 protein N-glycosylation and stability in HCC cells. To the best of our knowledge, we first provided the evidence that spermine can promote the expression of PD-L1 in HCC. Considering that elevated level of spermine in tumor tissues has been observed in patients with breast cancer, lung cancer or pancreatic cancer [60], we suppose that spermine can also promote tumor cell immune escape through inducing PD-L1 expression in these cancers. However, our study did not investigate whether spermine could induce the expression of PD-L1 in normal tissues, which is a limitation of this study. Recently, Li et al. found that the activation of epidermal growth factor receptor (EGFR) can inactivate GSK3β and thereby stabilizes PD-L1 expression, accounting for breast cancer cell immunosuppression [61]. Since hyperactivation of EGFR is frequently observed in hepatocellular carcinoma, which is associated with aggressive tumors and poor survival rates [62, 63]. Therefore, it raises the possibility that EGFR/GSK-3β/PD-L1 pathway may suppress T-cell activity in HCC.

Studies have suggested that N-linked glycosylation of PD-L1 could be catalyzed by STT3 isoforms or β-1,3-*N*-acetylglucosaminyltransferase (B3GNT3) [32, 64, 65]. Given that β-catenin was able to stabilize and upregulate PD-L1 through inducing STT3 isoforms (including A and B isoforms) in breast and colon cancers [32, 64], we therefore examined the roles of STT3 isoforms in spermine-induced PD-L1 *N*-glycosylation. Unexpectedly, our results suggested that only STT3A was involved in spermine-induced PD-L1 protein *N*-glycosylation and stability in hepatocellular carcinoma. This may be attributed to the different tumor microenvironment or the genetic background of the tumor entity. Although STT3A is critical for EMT-mediated PD-L1 protein induction and tumor immune evasion, the molecular mechanism through which STT3A is transcriptionally regulated in HCC cells remains unknown, we here disclosed that spermine could promote STT3A expression at transcriptional level through activation of Akt/β-catenin pathways. Furthermore, a variety of chemotherapy drugs such as cisplatin and c-MET inhibitors can induce the expression of PD-L1, generating drug resistance [66–68]. Therefore, it also raises the possibility that CaSR /AKT/β-catenin /PD-L1 pathway might play a critical role in the development of resistance.

## Conclusion

This study reveals for the first time that spermine promotes STT3A and PD-L1 expression through activation of Akt/β-catenin pathways, STT3A then stabilizes PD-L1 by modulating protein N-glycosylation in HCC. These results demonstrated that spermine exerts an immunosuppressive role through inducing the expression and *N*-glycosylation of PD-L1 in HCC, suggesting targeting of polyamine metabolism combined with PD-1/PD-L1 blockade immunotherapy may provide therapeutic benefits.

## Data Availability

The datasets used and/or analysed during the current study are available from the corresponding author on reasonable request.

## Abbreviations

ATG5: Autophagy-related 5
B3GNT3: β-1,3-*N*-acetylglucosaminyltransferase
CaSR: Calcium-sensing receptor
ChIP: Chromatin immunoprecipitation
EGFR: Epidermal growth factor receptor
GAPDH: Glyceraldehyde-3-phosphate dehydrogenase
GSK-3β: Glycogen synthase kinase-3β
GPCR: G protein-coupled receptor
HCC: Hepatocellular carcinoma
HPLC: High performance liquid chromatography
HRP: Horseradish peroxidase
IHC: Immunohistochemistry
IgG: Immunoglobulin G
LAK: Lymphokine-activated killer
myr-Akt1: Myristoylated Akt1
SDS-PAGE: Sodium dodecylsulfate-polyacrylamide gel electrophoresis
PBS: Phosphate-buffered saline
PSS: Physiological salt solution
PNGase F: *N*-glycosidase F
PD-L1: Programmed cell death-ligand 1
